# Identification and Immune Functional Characterization of Pigeon TLR7

**DOI:** 10.3390/ijms16048364

**Published:** 2015-04-14

**Authors:** Dan Xiong, Li Song, Zhiming Pan, Xiang Chen, Shizhong Geng, Xinan Jiao

**Affiliations:** 1 Jiangsu Co-innovation Center for Prevention and Control of Important Animal Infectious Diseases and Zoonoses, Yangzhou University, Yangzhou 225009, China; E-Mails: xiongdan.yzu@gmail.com (D.X.); songli.yzu@gmail.com (L.S.); chenxiang@yzu.edu.cn (X.C.); gszhong@yzu.edu.cn (S.G.); 2 Jiangsu Key Laboratory of Zoonosis, Yangzhou University, Yangzhou 225009, China

**Keywords:** TLR7, pigeon, identification, characterization, immune function

## Abstract

Toll-like receptor 7 (TLR7) is activated by single-stranded RNA and synthetic imidazoquinoline components, and induces interferon production. In this study, we cloned the *TLR7* gene from King pigeon (*Columba livia*). The *TLR7* open reading frame is 3144 bp and encodes a 1047-amino acid protein, consisting of a canonical TLR composition with 15 leucine-rich repeats (LRRs). Amino acid-inserting modifications were found at position 15 of LRR2, LRR11, LRR13, and LRR14 and position 10 of LRR10. The tissue distribution of pigeon TLR7 suggests that immune-associated tissues, especially the spleen and liver, have high TLR7 expression. HEK293T cells transfected with pigeon *TLR7* plasmid responded to the agonist R848, indicating a functional *TLR7* homolog. Following R848 stimulation of pigeon peripheral blood mononuclear cells, the levels of *IFN-γ*, *IL-6*, *IL-8*, *CCL5*, and *IL-10* mRNA, assessed using quantitative real-time PCR, were significantly up-regulated. After Newcastle disease virus vaccine strain LaSota inoculation and agonist R848 injection, the level of *TLR7* mRNA in the spleen of pigeons increased significantly in the R848-injected group, but decreased in the LaSota-inoculated group at three day post-infection (d.p.i.). The mRNA levels of inflammatory cytokines and chemokines were significantly upregulated in both LaSota-inoculated and R848-injected groups. Triggering pigeon *TLR7* leads to robust up-regulation of inflammatory cytokines and chemokines, suggesting an important role in the innate immune response.

## 1. Introduction

The innate immune system acts as the front line of defense against invading microorganisms in most multicellular organisms by recognizing pathogen-associated molecular patterns (PAMPs) through pattern recognition receptors (PRRs) [1]. Toll-like receptors (TLRs) form part of an ancient receptor family of PRRs and are the major cell-surface initiators of inflammatory responses to invading pathogens [2]. To date, 13 TLRs have been identified in mammals and 10 have been found in avian species, including the chicken, goose, and duck [3,4,5]. TLRs bind a wide variety of pathogen-associated substances through their ectodomains [6]. The basic framework of TLRs is a horseshoe-shaped solenoid, or “m”-shaped architecture that contains an extensive β-sheet on its concave surface, and numerous ligand-binding insertions [7]. These surfaces are responsible for ligand binding and ligand-induced TLR dimerization [8].

TLRs comprise a family of type I transmembrane receptors [9], which are composed of various leucine-rich repeats (LRRs), and are characterized by a signal peptide, an extracellular domain containing varying numbers of tandem LRRs, a *C*-terminal capping region overlapping the last LRR (LRR-CT), a transmembrane helix, and a cytoplasmic region containing a Toll interleukin-1 receptor domain [10,11].

Induction of the innate immune pathways is critical for early anti-viral defense, but there is limited understanding of how pigeons recognize viral molecules and activate these pathways. In mammals, TLR7 recognizes single-stranded RNA of viral origin and synthetic anti-viral imidazoquinoline compounds [12]. Upon activation, TLR7 recruits myeloid differentiation primary response protein 88 (MyD88) that, through several effector molecules, initiates the activation of two major signaling pathways resulting in the production of proinflammatory cytokines and/or type I interferons [13]. Collectively, these observations indicate that TLR7 plays a role in restricting the entry of viral pathogens in mammals. However, in birds, such as pigeons, *TLR7* has not yet been cloned and its function has not been reported.

In this paper, we describe the cloning and characterization of the pigeon *TLR7* sequence, analysis of its expression in various tissues, and evaluation of its function in response to the agonist R848 (resiquimod). Pigeon peripheral blood mononuclear cells (PBMCs) exposed to R848 stimulation showed significant induction of proinflammatory cytokine and interferon-γ (IFN-γ) production, but no significant change of the *TLR7* mRNA level *in vitro*. After Newcastle disease virus (NDV) inoculation and R848 injection of pigeons, the expression levels of proinflammatory cytokines and IFN-γ in spleen were significantly up-regulated and *TLR7* mRNA significantly increased in the R848-injected group, but significantly decreased in the LaSota-inoculated group at three day post-infection. These data expand our knowledge of the relationship between TLR7 and innate immunity in pigeons, which may be of relevance to other avian species, including goose and duck.

## 2. Results

### 2.1. Characterization of the Pigeon Toll-Like Receptor 7 (TLR7) Gene

**Figure 1:**
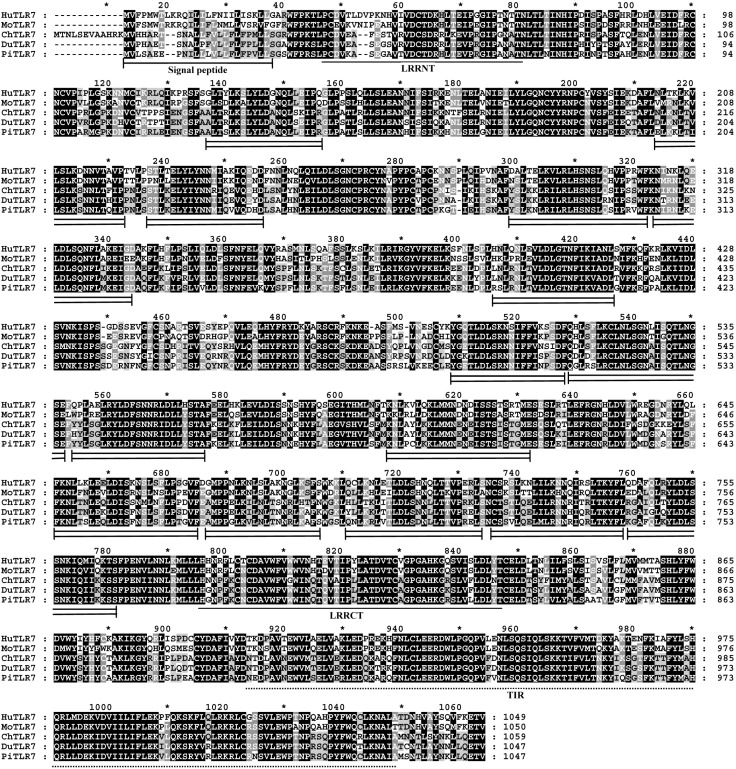
Alignment of TLR7 amino acid sequences. Hu, Mo, Ch, Du and Pi are short for human, mouse, chicken, duck and King pigeon (*Columba livia*) respectively. Sequences were aligned using ClustalW and edited using Genedoc. In the pigeon sequence, the predicted signal peptide and leucine-rich repeat *N*-terminal (LRR-NT) and *C*-terminal (LRR-CT) domains are underlined. The double continuous lines denote leucine-rich repeats, and the dotted line represents the Toll/IL-1 receptor (TIR) domain. Shading was performed using the conserved mode (black shading for conserved residues and light gray shading for similar residues). The asterisk symbol stands for the number of the amino acids. GenBank accession numbers for the aligned sequences: King pigeon (*Columba livia*) AIK67344, duck ABK51522, chicken CAF28878, human BAG55056, and mouse AAK62676.

The entire coding region of the *TLR7* gene from King pigeon (*Columba livia*) was successfully amplified from genomic DNA using primers overlaying the coding region. Sequencing results show the pigeon *TLR7* gene contains an open reading frame (ORF) of 3144 bp encoding a protein of 1047 amino acids (Figure 1). The sequence was submitted to GenBank and assigned the accession number KM086724.

Prediction of protein domains using the Simple Modular Architecture Research Tool (SMART; http://smart.embl-heidelberg.de) revealed that the putative amino acid sequence consisted of a signal peptide sequence encompassing the first 24 amino acid residues of the *N*-terminal region, an LRR-NT domain, 15 leucine-rich repeat (LRR) domains, an LRR-CT domain, and a 147-amino acid Toll interleukin-1 receptor domain at residues 888–1034 of the carboxy-terminus (Figure 1).

The deduced amino acid sequence of pigeon *TLR7* was aligned with reported sequences from human (GenBank ID: BAG55056), mouse (GenBank ID: AAK62676), chicken (GenBank ID: CAF28878), and duck (GenBank ID: ABK51522) using ClustalW. Multiple sequence alignment illustrated that the amino acid sequence of pigeon *TLR7* shared 65.0% identity with human, 62.2% with mouse, 81.5% with chicken, and 83.8% with duck (Figure 1).

**Figure 2:**
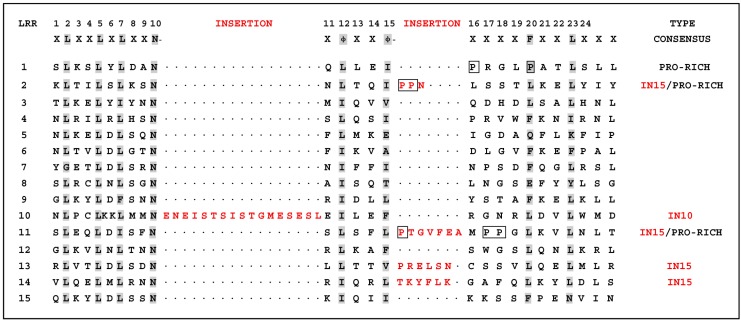
Alignments of selected leucine-rich repeats (LRRs) of pigeon TLR7 to the TLR consensus sequence. Gaps had been introduced into the TLR consensus sequence to allow for insertions in some of the LRRs. In the column marked TYPE, the short repeat with proline residues near the *C*-terminal end was designated PRO-RICH and the corresponding sequences were boxed, and LRRs with insertions at positions 10 and 15 were designated IN10 and IN15, respectively, and the corresponding sequences were shaded in red color.

The prevailing LRR consensus sequence for the TLRs is the 24-residue motif XLXXLXLXXNXφXXφXXXXFXXLX [10]. Sequence analysis of pigeon *TLR7* showed that it contains 15 regions containing this motif. However, several LRRs had developed amino acid insertion modifications compared with the general motif. In particular, insertion of various amino acids occurs at position 15 of LRR2, LRR11, LRR13, and LRR14. Notably different from other repeats is LRR10, which contains long insertions at position 10 following the consensus Asn residue. Meanwhile, a repeat with a proline-rich *C*-terminal portion occurs in LRR1, LRR2, and LRR11 at position 15 (Figure 2).

### 2.2. Sequence Analysis of TLR7

**Figure 3:**
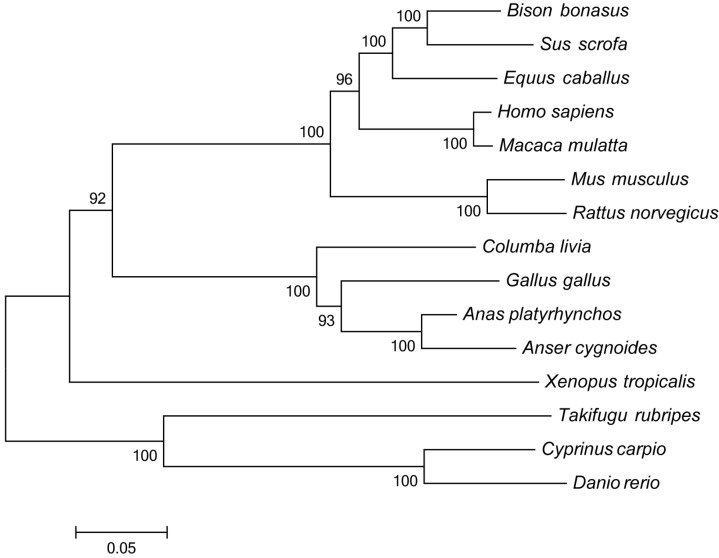
Phylogenetic tree of the TLR7 amino acid sequences from different species. The tree was constructed by the neighbor-joining method based on the Poisson correction model with 1000 bootstrap replicates using MEGA 5.1 software. The numbers at the nodes indicate bootstrap values. The bar (0.05) indicates the genetic distance. GenBank accession numbers: *Columba livia* AIK67344, *Gallus gallus* CAF28878, *Anas platyrhynchos* ABK51522, *Anser cygnoide*s AHM88222, *Bison bonasus* ACA34988, *Sus scrofa* ABO09808, *Equus caballus* NP_001075240, *Homo sapiens* BAG55056, *Macaca mulatta* NP_001123898, *Mus musculus* AAK62676, *Rattus norvegicus* NP_001091051, *Xenopus tropicalis* AAI66280, *Takifugu rubripes* AAW69375, *Cyprinus carpio* AB553573, and *Danio rerio* XP_003199309.

Phylogenetic analyses were performed on the amino acid sequences of the full coding region of TLR7 using the neighbor-joining method. The resulting phylogenetic tree, consisting of 15 protein sequences, is composed of four major branches (Figure 3). TLR7 protein sequences from the avian species *Columba livia* (GenBank ID: AIK67344), *Gallus gallus* (GenBank ID: CAF28878), *Anas platyrhynchos* (GenBank ID: ABK51522), and *Anser cygnoides* (GenBank ID: AHM88222) were in the same subgroup as the sequences from the mammals *Bison bonasus* (GenBank ID: ACA34988), *Sus scrofa* (GenBank ID: ABO09808), *Equus caballus* (GenBank ID: NP_001075240), *Homo sapiens* (GenBank ID: BAG55056), *Macaca mulatta* (GenBank ID: NP_001123898), *Mus musculus* (GenBank ID: AAK62676), and *Rattus norvegicus* (GenBank ID: NP_001091051). *Xenopus tropicalis* (GenBank ID: AAI66280) constituted the single branch of batrachian. The piscine TLR7 protein sequences, including *Takifugu rubripes* (GenBank ID: AAW69375), *Cyprinus carpio* (GenBank ID: AB553573), and *Danio rerio* (GenBank ID: XP_003199309), were in another subgroup. The pigeon, chicken, duck, and goose TLR7 sequences were the most closely related, as expected. The observed relationships within this cluster reflected the taxonomic positions of these species.

### 2.3. Expression of Pigeon TLR7 mRNA in Different Tissues

**Figure 4:**
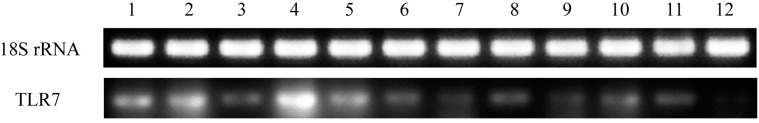
Expression analysis of the pigeon *TLR7* gene in various tissues by semiquantitative RT-PCR. Total RNA was extracted from different tissues of a healthy King pigeon (*Columba livia*) and reverse transcribed to cDNA. 18S rRNA was amplified as the endogenous control. 1, peripheral blood mononuclear cells (PBMCs); 2, spleen; 3, neck lymph nodes; 4, liver; 5, lung; 6, kidney; 7, heart; 8, bone marrow; 9, small intestine; 10, large intestine; 11, cecum; 12, brain.

To investigate the expression of *TLR7* mRNA in normal pigeon tissues, a semi-quantitative RT-PCR analysis was performed. As shown in Figure 4, the pigeon *TLR7* gene was highly expressed in PBMCs, spleen, liver, and lung; moderately expressed in neck lymph nodes, kidney, bone marrow, large intestine, and cecum; and minimally expressed in the heart, small intestine, and brain.

### 2.4. Immunoblotting

**Figure 5:**
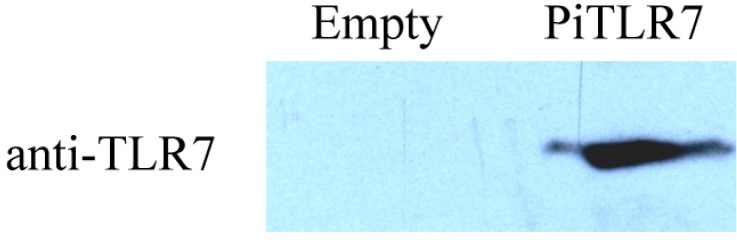
Immunoblotting of anti-TLR7 monoclonal antibody. The HEK293T cells transfected with empty vector (Lane 1) or plasmid pCMV-PiTLR7 (pCMV-PigeonTLR7) (Lane 2) were harvested and then the protein was subjected to Western blotting analysis using the anti-TLR7 monoclonal antibody.

To verify the expression of *TLR7 in HEK293T cells transfected with pCMV-PiTLR7* (pCMV-PigeonTLR7) plasmid, an anti-TLR7 monoclonal antibody raised against a partial recombinant TLR7 was used for Western blotting. The result showed that pigeon TLR7 was successfully expressed in PiTLR7-transfected cells. In contrast, the protein from control cells bearing the empty vector did not react with the same antibody (Figure 5).

### 2.5. Functional Analysis of Pigeon TLR7

**Figure 6:**
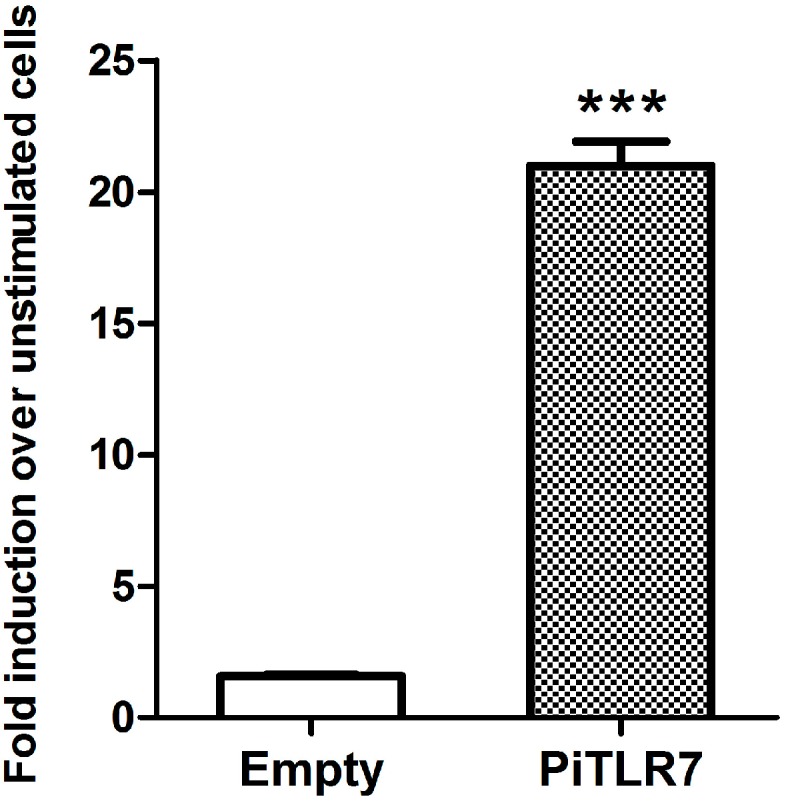
Effect of R848 (resiquimod)-stimulation on NF-κB activity based on firefly luciferase (FLU). HEK293T cells were transfected with an expression vector (pCMV-PiTLR7 or pCMV-empty) and a reporter vector (pGL4.32(luc2P/NF-κB-RE/Hygro)). Twenty-four hours post-transfection, 2.5 μg/mL of R848 was added to the transfected cells and the induction levels of NF-κB luciferase activity were measured after stimulation for 5 h. Columns represent the mean ± SD of three independent experiments. Asterisks indicate significant differences by a *t*-test between pigeon TLR7- and empty vector-transfected cells (*p* < 0.001).

To evaluate the response of pigeon TLR7 to R848, the effect of R848 stimulation on NF-κB activity was determined. The gene was cloned into the pCMV expression vector and the recombinant pCMV-PiTLR7 plasmid was transfected into HEK293T cells. NF-κB-induced luciferase activity was then assayed following R848 stimulation at a concentration of 2.5 μg/mL. As shown in Figure 6, the induction level of luciferase activity in *TLR7*-transfected cells 5 h after stimulation was 20-fold higher than that in cells transfected with empty vector control (*p* < 0.001).

### 2.6. Induction of Inflammatory Cytokines and Chemokines in R848 (Resiquimod)-Stimulated Peripheral Blood Mononuclear Cells (PBMCs)

**Figure 7:**
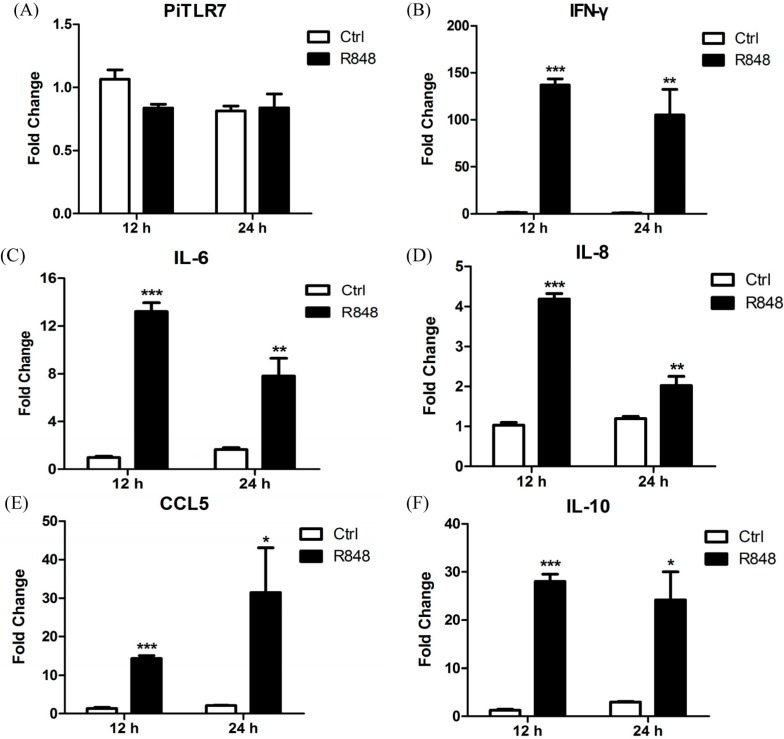
Levels of *TLR7* (**A**); *IFN-γ* (**B**); *IL-6* (**C**); *IL-8* (**D**); *CCL5* (**E**); and *IL-10* (**F**) mRNAs in pigeon PBMCs following R848 stimulation. Pigeon peripheral blood mononuclear cells (PBMCs) were isolated from whole blood of healthy King pigeons. Cells were stimulated with a TLR7 agonist R848 for 12 and 24 h respectively. Data shown are the fold changes in mRNA expression compared with control based on triplicate repeats and determined by qRT-PCR. Error bars indicate standard deviations of the means. Statistical significance was determined at *p* < 0.05 (*), *p* < 0.01 (**) or *p* < 0.001 (***).

Quantitative analysis of IFN-γ expression at 12 and 24 h post injection (h.p.i.) indicated 140- and 100-fold increases respectively, compared with medium-treated controls. The mRNA expression of the inflammatory cytokine IL-6 was significantly upregulated at 12 h.p.i. (13-fold) and then slightly decreased at 24 h.p.i. (7-fold). The expression of chemokines IL-8 and CCL5 showed a 4-fold and 14-fold up-regulation respectively at 12 h.p.i., followed by a slightly reduced expression of IL-8 (2.7-fold) and a sustained increase of CCL5 (30-fold) at 24 h.p.i.. Interestingly, the mRNA expression of the anti-inflammatory cytokine IL-10 was significantly upregulated at 12 h.p.i. (30-fold) and 24 h.p.i. (24-fold). The expression level of PiTLR7 was not statistically significantly different compared with controls at either time point (Figure 7).

### 2.7. Effect of Newcastle Disease Virus (NDV) Vaccine Strain LaSota and Agonist R848 on the Expression of Pigeon TLR7 and Inflammatory Cytokines

**Figure 8:**
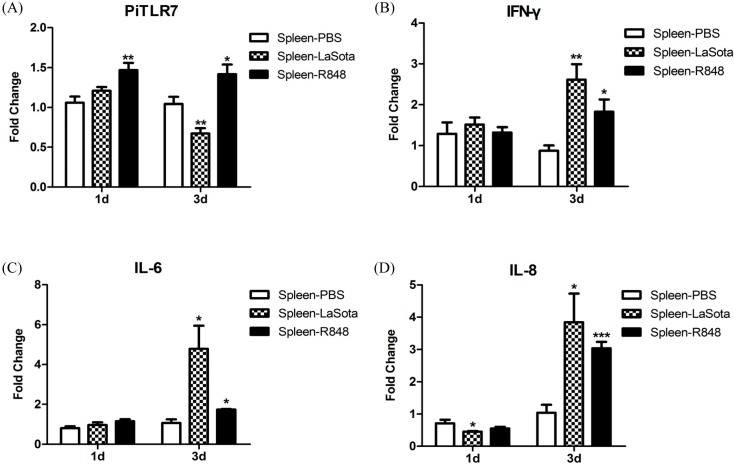

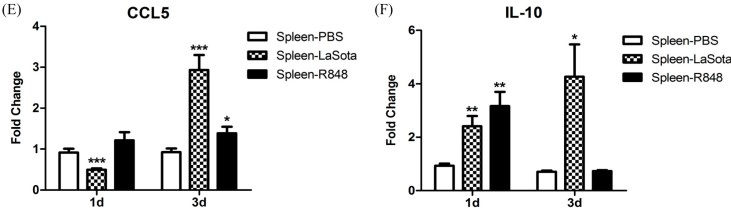
Levels of *TLR7* (**A**); *IFN-γ* (**B**); *IL-6* (**C**); *IL-8* (**D**); *CCL5* (**E**); and *IL-10* (**F**) mRNAs in spleens of pigeons following LaSota inoculation or R848 injection. King pigeons were injected in an intramuscular route either with 10 μg R848, 10^6^ mean egg infectious dose (EID_50_) LaSota or phosphate-buffered saline (PBS) in 200 μL respectively. Data shown are the fold changes in mRNA expression compared with PBS control at 1 and 3 day post infection, based on six pigeons for each time and determined by qRT-PCR. Error bars indicate standard deviations of the means. Statistical significance was determined at *p* < 0.05 (*), *p* < 0.01 (**) or *p* < 0.001 (***).

To characterize the effect of the NDV vaccine strain LaSota and the TLR7 agonist R848 on the induction of host responses, the mRNA expression levels of pigeon *TLR7* and cytokines in the spleen were detected by quantitative real-time PCR (Figure 8). Pigeon *TLR7* gene expression in spleen of pigeons injected with R848 was slightly but significantly increased (1.4-fold) over the control pigeons 1 and 3 days post-infection (d.p.i.). In contrast, *TLR7* expression in the LaSota-inoculated group was significantly decreased (0.7-fold) 3 d.p.i when compared with the control group. The antiviral cytokine IFN-γ showed no significant difference in the LaSota-inoculated or R848-injected groups compared with the control group at 1 d.p.i., but was elevated significantly in the LaSota-inoculated group (2.5-fold) and R848-injected group (1.8-fold) at 3 d.p.i. The expression of the inflammatory cytokine IL-6 showed the same tendency as that of IFN-γ, with no significant induction between groups at 1 d.p.i and significant increases in the LaSota-inoculated and R848-injected groups at 3 d.p.i. (4.5- and 1.8-fold, respectively). A significant reduced expression of the chemokines IL-8 and CCL5 (0.6- and 0.5-fold, respectively) were found in the LaSota-inoculated, but not the R848-injected, group at 1 d.p.i., while both of these chemokines were significantly upregulated in the LaSota-inoculated group (3.7- and 2.8-fold, respectively) and the R848-injected group (3.0- and 1.5-fold, respectively) at 3 d.p.i. The anti-inflammatory cytokine IL-10 was significantly induced in the LaSota-inoculated and R848-injected groups (2.5- and 3.2-fold, respectively) at 1 d.p.i.; a sustained increase was found in the LaSota-inoculated group (4.0-fold), but this reverted back to the original level in the R848-injected group at 3 d.p.i.

## 3. Discussion

Among the PRRs, TLRs are crucial for recognition of pathogen-derived products, and initiate signaling cascades leading to the activation of innate host defenses [14]. In contrast to mammalian TLR7 orthologs, little is known about the sequence variation and biological function of avian TLR7. The demonstrated response of duck and goose TLR7 to imidazoquinoline suggests a similar role for avian TLR7 homologs in the immune response of birds [15,16].

LRRs are found in a diverse set of proteins in which they are involved in ligand recognition and signal transduction [17]. The structures of LRRs in TLRs are speculated to mediate recognition of specific PAMPs [7]. Current evidence indicates that these irregular LRRs are involved in PAMP recognition, and a residue insertion after position 15 in LRR14 of human TLR5 contributes to flagellin binding [18]. Interestingly, among the 15 LRR domains in pigeon TLR7 with the prevailing LRR motif XLXXLXLXXNXφXXφXXXXFXXLX [10,19], five LRRs differ from the canonical sequence by amino acid insertions, including LRR2, LRR11, LRR13, and LRR14 at position 15, and LRR10 at position 10. A *C*-terminal proline-rich LRR, similar to some bacterial LRRs [7], was found in LRR1, LRR2 and LRR11 at position 15 of pigeon TLR7 (Figure 2). The most striking modifications to the TLR consensus are large insertions that occur at positions 10 or 15, and insertions or other irregularities on the convex surface might affect PAMP binding by introducing flexibility into the TLR horseshoe [7]. Thus, the insertions in the LRRs of pigeon TLR7 may influence its functional role as a pathogen receptor and further studies are needed to investigate this.

The gene expression patterns of *TLR7 in healthy pigeon tissues were assessed in this study. TLR7* was expressed in all tissues examined, although expression levels varied (Figure 4). This is consistent with the finding that duck *TLR7* is expressed in a broad range of tissues and cell types [15]. In this study, high expression levels of pigeon *TLR7* mRNA were observed in the PBMCs, spleen, liver, and lung, whereas the lowest expression was observed in the heart, small intestine, and brain. The expression profile of pigeon *TLR7* differs from that of goose *TLR7*, which displays high expression levels in the cecum and lung, but low expression in the liver and kidney [16]. These findings illustrate species-based differences in the tissue distribution of *TLR7* expression. The observation that pigeon *TLR7* is expressed at sites of viral contact in the lung and immune organs is consistent with a role in the host innate immune system as the first line of defense against pathogens in these tissues.

Imidazoquinoline is a TLR7 ligand [20] that upon receptor binding with TLR7, induces translocation of NF-kB and production of TNF-α, IL-6, IL-12, and type I interferon [12,21]. *In vitro* experiments indicate that immunostimulatory RNA oligonucleotides (ORN) found in foot-and-mouth disease virus can activate nuclear factor-κB via porcine TLR7 [22]. In this study, following stimulation with R848, the induction level of NF-κB-induced luciferase activity in pigeon *TLR7*-transfected HEK293T cells was significantly higher than that in cells transfected with an empty vector control (Figure 6). Our results suggest that pigeon TLR7 is a pathogen receptor that plays a role in the recognition of the agonist R848.

Goose spleen mononuclear cells infected by New type gosling viral enteritis virus (NGVEV) *in vitro* were unable to increase the expression of TLR7. Instead, levels of TLR7 in the spleen of NGVEV-infected geese increased dramatically [16]. Similar to goose TLR7, the mRNA expression of pigeon *TLR7* was not statistically significantly different compared with controls following R848 stimulation of pigeon PBMCs (Figure 7). However, *TLR7* gene expression in the spleen of pigeons injected with R848 was slightly but significantly increased over the controls (Figure 8). Another study demonstrated that both duck and chicken *TLR7* are only transiently expressed in PBMCs at the early stages of low pathogenic avian influenza virus H11N9 infection, followed by a decline as the infection progresses [23]. In variant strain infectious bursal disease virus-infected bursa, TLR7 expression was downregulated at 3 and 5 d.p.i. and upregulated at 7 d.p.i. [24]. In this study, TLR7 expression in the LaSota-inoculated group appeared to be slightly elevated at 1 d.p.i., but this was not statistically significant, and significantly decreased 3 d.p.i. when compared with the control (Figure 8).

TLR7 plays an essential role in inflammatory cytokine activation in granulocyte-macrophage colony-stimulating factor (GM-CSF)-primed murine neutrophils in response to influenza and the R848, as has been shown using *TLR7^−/−^* mice. Murine *TLR7^−/−^* neutrophils had poor responses to both R848 and influenza virus in comparison with wild-type cells [25]. In this study, following R848 stimulation of pigeon PBMCs, the expression levels of the anti-viral cytokine IFN-γ, and of inflammatory cytokines, were robustly upregulated at 12 and 24 h.p.i. Interestingly, the anti-inflammatory cytokine IL-10 was also significantly increased at both time points (Figure 7) and further studies are needed to ascertain the reason for this observation. In response to LaSota inoculation or R848 injection of pigeons, the mRNA levels of *IFN-γ*, *IL-6*, *IL-8*, *CCL5*, and *IL-10* in the spleen were markedly elevated (Figure 8). An *in vivo* study by Rasoli *et al.* reported that significant upregulation in the mRNA expression level of IL10 correlated with downregulation of the mRNA levels of proinflammatory cytokines and chemokines in AF2240 and IBS002 strains of NDV-infected spleen at 4 d.p.i. [26]. Notably, we observed that the level of the anti-inflammatory cytokine IL-10 was dramatically reduced (to the original level in the R848-injected group) to 3 d.p.i., which was concomitant with the upregulation of inflammatory cytokines (Figure 8). R848 stimulation or LaSota inoculation resulted in the rapid up-regulation of proinflammatory cytokines and anti-viral molecules, suggesting that pigeon TLR7 plays an important role in the innate immune response.

## 4. Experimental Section

### 4.1. Molecular Cloning of Pigeon TLR7

King pigeons (*Columba livia*) purchased from Jiangyin Wei Tekai Pigeon Co. (Wuxi, China) were housed in isolators and fed with a pathogen-free diet and water. The procedures described in this study were approved by the Ethics Committee on Animal Experiments of Yangzhou University, Yangzhou, China (Yangzhou University, [2012] no. 62, 12 December 2012). Genomic DNA was extracted from whole blood of the pigeons using a Universal Genomic DNA Extraction Kit Ver. 3.0 (Takara Biotechnology Co., Dalian, China) according to the manufacturer’s instructions. To clone the pigeon *TLR7* gene, primer pairs covering the entire open reading frame (ORF) were designed based on the predicted pigeon nucleotide (GenBank ID: XM_005512700) and the well-conserved upstream and downstream sequences of *TLR7* from chicken (*Gallus gallus*; GenBank ID: NM_001011688) and duck (*Anas platyrhynchos*; GenBank ID: DQ888645). PCR was performed using the designed primers (Table 1) and pigeon genomic DNA as a template. PCR was carried out using PrimeSTAR HS DNA Polymerase (Takara Biotechnology Co.) in a 50-μL reaction volume consisting of 1× PrimeSTAR Buffer (Mg^2+^ plus), 200 µM of each deoxy-ribonucleoside triphosphate (dNTP), 0.2 µM of forward and reverse primers, 1.25 U of PrimeSTAR Hot Start (HS) DNA Polymerase, and ~200 ng of genomic DNA template. PCR amplifications were performed as follows: 1 cycle of 98 °C for 5 min, then 30 cycles of 98 °C for 10 s, 60 °C for 15 s, and 72 °C for 3 min, followed by 1 cycle of 72 °C for 10 min. The amplified PCR product was purified, cloned into pCR2.1-T using a TA Cloning Kit (Invitrogen, Carlsbad, CA, USA), and sequenced by Genscript (Nanjing, China).

**Table 1:** PCR primers used in this study. The underlined parts in PiTLR7 F/R represent the introduced *Sal*I and *Kpn*I restriction enzyme sites.

Primer Name	Primer Sequence (5'→3')	Size (bp)	Application
PiTLR7 F	ACGCGTCGACCATGGTACTTAGTGCAGAAGAGCCAAATAC	3144	Amplification of Pigeon TLR7 ORF
PiTLR7 R	CCCCGGTACCCTAAACAGTTTCTTGGAGAAGCTTGTTG		
PiTLR7-RT-F	CAGACTCAAGTGACTATTCCTCTTCTG	216	RT-PCR
PiTLR7-RT-R	GTAACTATACCACACATCCCAGAAATAGAG		
18S rRNA F	TTGGTGGAGCGATTTGTC	129	RT-PCR
18S rRNA R	ATCTCGGGTGGCTGAACG		
Piβ-actin F	ATGAAGCCCAGAGCAAAAGAG	223	Quantitative real-time PCR
Piβ-actin R	GGGGTGTTGAAGGTCTCAAAC		
PiTLR7 F	ACCAGCGGCTTCTAGATGAA	158	Quantitative real-time PCR
PiTLR7 R	CTGCCAAAAGTAGGGCTGAG		
PiIFN-γ F	CAGACGTAGCTGATGGTGGAC	233	Quantitative real-time PCR
PiIFN-γ R	AAGCTTTGCCAGATCCTTGAG		
PiIL-6 F	AGCGTCGATTTGCTGTGCT	107	Quantitative real-time PCR
PiIL-6 R	GATTCCTGGGTAGCTGGGTCT		
PiIL-8 F	CTGTCCTGGCTCTTTTCCTG	199	Quantitative real-time PCR
PiIL-8 R	CTGCCGTCCTTCAGAGTAGC		
PiCCL5 F	GTGAAGGACTATTTCTACACCAGCA	95	Quantitative real-time PCR
PiCCL5 R	GCGTCAGGGTTTGCACAGA		
PiIL-10 F	TGATGAACTTAGCATCCAGCTACTC	93	Quantitative real-time PCR
PiIL-10 R	AACTGCATCATCTCCGACACA		

### 4.2. Sequence Analyses

The nucleotide and deduced amino acid sequences of pigeon *TLR7* were analyzed using DNAstar software and the Expert Protein Analysis System (Expasy, http://www.mrc-lmb.cam.ac.uk/genomes/madanm/pres/swiss1.htm). SMART was used to predict the protein domain structure of pigeon *TLR7*. *TLR7* sequences from different species were compared using the NCBI BLAST search program (http://blast.ncbi.nlm.nih.gov/Blast.cgi). A multiple sequence alignment was performed using ClustalW (http://www.ebi.ac.uk/clustalw/) and edited with the Genedoc program. Phylogenetic analysis was conducted on amino acid sequences using MEGA 5.1 software (http://www.megasoftware.net), and a phylogenetic tree was constructed with the neighbor-joining method using a Poisson correction model with 1000 bootstrap replicates.

### 4.3. Semi-Quantitative Analysis of Pigeon TLR7 Expression in Tissues

Total RNA was extracted from peripheral blood mononuclear cells (PBMCs), spleen, neck lymph nodes, liver, lung, kidney, heart, bone marrow, small intestine, large intestine, cecum, and brain of healthy King pigeons using TRIzol reagent (Invitrogen), according to the manufacturer’s instructions. Total RNA samples were treated with RNase-Free DNase I (Takara Biotechnology Co.) to remove contaminating DNA, and first-strand cDNA was synthesized using M-MLV reverse transcriptase (Invitrogen) following the manufacturer’s protocol. RT-PCR was performed with cDNA templates from each tissue and a specific primer set for *TLR7* (Table 1). The endogenous control was 18S rRNA from pigeon (GenBank ID: AF173630). PCR products were run on 1% (*w*/*v*) agarose gels.

### 4.4. DNA Constructs

An expression construct, pCMV-TLR7, was made by cloning the full-length pigeon *TLR7* coding sequence into the *Sal*I and *Kpn*I sites of the pCMV-HA-tag expression vector (Invitrogen). Plasmid pCR2.1-TLR7, containing the full-length TLR7 open reading frame (ORF), was digested with *Sal*I and *Kpn*I (Takara Biotechnology Co.), and subcloned into the same restriction enzymes sites of pCMV-HA. The resulting construct was designated pCMV-PiTLR7.

### 4.5. Cell Culture, Transfection and Stimulation

HEK293T cells were grown in 24-well tissue culture plates in Dulbecco’s Modified Eagle’s Medium (DMEM) supplemented with 10% fetal bovine serum (FBS) until 70% confluence was reached. Cells were washed with PBS before transfection, and the medium was then replaced with Opti-MEM (Invitrogen) medium. Transient transfection was performed using Lipofectamine 2000 (Invitrogen) according to the manufacturer’s instructions. Equal amounts of DNA constructs, including pCMV-TLR7 or pCMV-empty, and 400 ng of reporter pGL4.32 (luc2P/NF-κB-RE/Hygro) plasmid DNA (Promega, Madison, WI, USA) were transfected into HEK293T cells. Twenty-four hours after transfection, endotoxin-free R848 (Enzo Life Sciences, Farmingdale, NY, USA) was added to a final concentration of 2.5 μg/mL. After stimulation for 5 h, cells were harvested for subsequent western blotting or Luciferase assay.

### 4.6. Western Blotting Analysis

Harvested cells were subjected to SDS-PAGE and the proteins were transferred to a nitrocellulose membrane. The membrane was blocked with blocking buffer (5% non-fat dry milk and 0.05% Tween-20 in PBS) at 4 °C overnight. The next day, the membrane was incubated with an anti-TLR7 monoclonal antibody raised against a partial recombinant human TLR7 (NP_057646, 27~126 amino acids, sharing 77% similarity with pigeon TLR7) (1:800 diluted in PBS-0.05% Tween 20, Abnova Corporation, Walnut, CA, USA) at 37 °C for 2 h. After washing three times with PBS-0.05% Tween 20 (PBST), the membrane was incubated with a horseradish peroxidase-conjugated secondary antibody (1:5000 diluted in PBST, Sigma, St. Louis, MO, USA) at 37 °C for 1 h. The protein bands were visualized using an enhanced chemiluminescence reagent (Thermo, Rockford, IL, USA).

### 4.7. Luciferase Assay

To determine the functional response of pigeon TLR7 to R848 stimulation, NF-κB-induced luciferase activity was measured using the Bright-Glo Luciferase Assay system (Promega, Madison, WI, USA) according to the manufacturer’s instructions.

### 4.8. Pigeon PBMCs Preparation and Stimulation

PBMCs were isolated from whole blood as previously described [27]. Briefly, the blood sample was collected with 10% EDTA at a 10:1 (*v*:*v*) ratio from healthy King pigeons and centrifuged for 10 min at 200× *g*. The erythrocyte pellet was resuspended in PBS, then layered onto 1077 Histopaque (Sigma) and centrifuged at 400× *g* for 30 min. Mononuclear cells were collected from the gradient interface, washed with PBS, and centrifuged for 10 min at 200× *g*. A final wash was performed with antibiotic-free RPMI 1640 (Invitrogen) supplemented with 10% fetal bovine serum (FBS) (Invitrogen). The pellets were then resuspended in antibiotic-free RPMI 1640 with FBS. Cell viability and number were determined by trypan blue exclusion. Pigeon PBMCs were cultured in RPMI 1640 medium containing 2% FBS, 1% penicillin/streptomycin, and 2 mM l-glutamine. After plating, cells were incubated overnight at 41 °C and then washed with PBS to remove non-adherent cells. Cells were stimulated with the TLR7 agonist R848 at a concentration of 2.5 μg/mL for either 12 or 24 h, then harvested for subsequent mRNA detection of immune-related genes.

### 4.9. Virus Inoculation and R848 Injection of Pigeons

King pigeons were housed and handled following approval by the Institutional Animal Experimental Committee (Yangzhou University, [2012] no. 62, 12 December 2012). Pigeons were randomly divided into three groups (six pigeons per group) and injected via an intramuscular route with either 10 μg R848 (Enzo Life Sciences, Farmingdale, NY, USA), 10^6^ EID_50_ of LaSota (Wuhan Chopper Biology Co., Wuhan, China), or PBS in 200 μL. At 1 and 3 d.p.i., pigeons were sacrificed by pentobarbital overdose, and the spleens were removed and stored at −70 °C for subsequent mRNA analysis of immune-related genes.

### 4.10. RNA Isolation, RT-PCR and Quantitative Real-Time PCR

Harvested cells or spleens were homogenized in TRIzol reagent (Invitrogen), and total RNA was prepared as described by the manufacturer. RNA concentrations were determined by spectrophotometer readings at 260 nm. Quantitative real-time PCR (qRT-PCR) was performed to measure mRNA expression levels of *TLR7*, *IFN-γ*, *IL-6*, *IL-8*, *CCL5*, and *IL-10* using SYBR Premix Ex Taq II (Perfect Real Time; Takara Biotechnology Co.) using an ABI 7500 real-time detection system (Applied Biosystems, Carlsbad, CA, USA) with designed primers (Table 1). Amplification was performed in a total volume of 20 μL containing 10 μL of 2× SYBR Premix Ex Taq II, 2 μL of the diluted cDNA, and 0.8 μL of each primer. The real-time PCR program started with denaturing at 95 °C for 30 s, followed by 40 cycles of 95 °C for 5 s and 60 °C for 34 s. Dissociation analysis of amplification products was performed at the end of each PCR to confirm that only one PCR product was amplified and detected. Data were analyzed using ABI 7500 SDS software (Applied Biosystems, Foster, CA, USA), with the baseline being set automatically by the software. The threshold method was used for quantification of the mRNA level [28] and Δ*C*_t_ values were calculated on the basis of the internal standard (ACTB, actin-beta) signal. Results are expressed as 2^−ΔΔ*C*t^ (n-fold change compared to the control group).

### 4.11. Statistical Analysis

The significance of the differences in the experimental data between *TLR7*-positive and empty vector-transfected groups, or between R848-stimulated/LaSota-inoculated and control groups, were determined using the Student’s *t*-test with Instat version 5.0 (GraphPad Software, San Diego, CA, USA). Statistical significance was determined at *p* < 0.05 (*), *p* < 0.01 (**) or *p* < 0.001 (***).

## 5. Conclusions

We have cloned and sequenced the *TLR7* gene from the genomic DNA of King pigeon (*Columba livia*). We characterized its predicted protein domains and determined that its mRNA is broadly expressed in most tissues. We also identified some insertions at positions 10 and 15 in LRRs and ascertained the function of pigeon TLR7 in response to R848 stimulation. R848 stimulation or LaSota inoculation resulted in the rapid up-regulation of proinflammatory cytokines and anti-viral molecules, suggesting that pigeon TLR7 plays an important role in the innate immune response. Additional studies to dissect the functional differences of amino acid insertions in LRRs, and the decrease of pigeon TLR7 expression following LaSota inoculation, may provide deeper insights into the structure and biological function of pigeon TLR7.
